# RETRACTED: Kaur et al. Synthesis of CaFe_2_O_4_-NGO Nanocomposite for Effective Removal of Heavy Metal Ion and Photocatalytic Degradation of Organic Pollutants. *Nanomaterials* 2021, *11*, 1471

**DOI:** 10.3390/nano16201299

**Published:** 2026-10-14

**Authors:** Manmeet Kaur, Manpreet Kaur, Dhanwinder Singh, Aderbal C. Oliveira, Vijayendra Kumar Garg, Virender K. Sharma

**Affiliations:** 1 Department of Chemistry, Punjab Agricultural University, Ludhiana 141001, Punjab, India; manmeetgill885@gmail.com; 2 Department of Soil Science, Punjab Agricultural University, Ludhiana 141001, Punjab, India; dhanwinder@pau.edu; 3 Institute of Physics, University of Brasilia, Brasilia 70000-000, Brazil; aderbal47@gmail.com (A.C.O.); vijgarg@gmail.com (V.K.G.); 4 Program for Environment and Sustainability, Department of Environmental and Occupational Health, School of Public Health, Texas A&M University (TAMU), College Station, TX 77843-1266, USA

The Journal retracts the article titled, “Synthesis of CaFe_2_O_4_-NGO Nanocomposite for Effective Removal of Heavy Metal Ion and Photocatalytic Degradation of Organic Pollutants” [1], cited above.

Following the publication of a correction to this article [1], Texas A&M University informed the Editorial Office of the outcome of an institutional investigation concerning the integrity of the XRD data presented in Figure 1 of the original article [1].

In accordance with the journal’s standard procedures, the Editorial Office and Editorial Board conducted a further investigation, which confirmed the findings of the institutional investigation. Although the authors cooperated throughout the process, they did not inform the Editorial Office of the ongoing institutional investigation at the time the correction was published. The Editorial Board concluded that the concerns regarding the XRD data could not be adequately addressed through the published correction. As Figure 1 was considered central to the article’s findings, and because the supporting materials provided were insufficient to verify the validity of the data, the Editorial Board determined that confidence in the integrity and reliability of the published results could no longer be maintained.

As a result, the Editorial Board has decided to retract this article in accordance with MDPI’s retraction policy. This decision is consistent with the outcome of the investigation conducted by Texas A&M University.

This retraction was approved by the Editor-in-Chief of *Nanomaterials*.

The authors have been informed of this retraction and have indicated their disagreement with this decision.
